# Laparoscopic removal of a fishbone migrating from the gastrointestinal tract to the pancreas

**DOI:** 10.1002/ccr3.3822

**Published:** 2021-01-19

**Authors:** Francesk Mulita, Dimitris Kehagias, Levan Tchabashvili, Fotis Iliopoulos, Nikolas Drakos, Ioannis Kehagias

**Affiliations:** ^1^ Department of General Surgery General University Hospital of Patras Patras Greece

**Keywords:** fish bone, foreign body, laparoscopic surgery, pancreas

## Abstract

Laparoscopic surgery can be performed safely for the removal of a foreign body embedded in the pancreas and should be preferred instead of open surgery, whenever possible.

## Graphical abstract

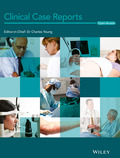

## 1 CASE DESCRIPTION

A 59‐year‐old female patient presented to the emergency department with epigastric pain. On examination, she had no significant findings. Computed tomography of the abdomen showed a linear, hyperdense, foreign body between the prepyloric region of the stomach and the pancreatic head. Although the foreign body had perforated the posterior wall of the prepyloric region of the stomach, the patient had no signs of peritonitis, since there was no communication between the gastric lumen and the peritoneal cavity. Emergency laparoscopic surgery was performed. The foreign body was safely removed laparoscopically and was identified as a 3‐cm‐long fishbone (Video S1). Very few cases of an ingested fishbone that migrated into the pancreas have been published in the literature so far.^1^, ^2^ The patient recovered without complications and was discharged. Laparoscopic surgery could be performed safely for the removal of an ingested foreign body embedded in the pancreas.

## CONFLICT OF INTEREST

None declared.

## AUTHOR CONTRIBUTIONS

FM, DK, LT, FI, and ND: contributed to the clinical data collection and prepared the case report. FM and IK: contributed to the design of the case report presentation and performed the final revision of the manuscript.

## PATIENT CONSENT FOR PUBLICATION

A written informed consent was obtained from the patient for publication of this case report.

## Supporting information

Video S1

## Data Availability

Data available on request from the authors.
